# Building blocks of functional connectivity measures for aperiodic electrophysiological brain signals

**DOI:** 10.1016/j.ynirp.2025.100284

**Published:** 2025-09-13

**Authors:** Rikkert Hindriks, Thomas O. Rot, Michel J.A.M. van Putten, Prejaas Tewarie

**Affiliations:** a Department of Mathematics, Faculty of Science, Vrije Universiteit Amsterdam, De Boelelaan 1105, 1081 HV Amsterdam, The Netherlands; b Clinical Neurophysiology Group, University of Twente, Drienerlolaan 5, 7522 NB Enschede, The Netherlands; c Sir Peter Mansfield Imaging Center, School of Physics, University of Nottingham, University Park, Nottingham, NG7 2RD, United Kingdom; d Joint International Research Unit on Neuroplasticity, CERVO Brain Research Centre, Laval University, 2601, de la Canardière Québec, G1J 2G3, Canada

**Keywords:** Electroencephalography, Functional connectivity, Volume conduction, Aperiodic signal, Local field potential, Utah array, Plücker coordinate, Grassmann manifold

## Abstract

A challenge in interpreting functional connectivity results in electroencephalography (EEG) data is volume conduction. A common way to mitigate spurious connectivity due to volume conduction is to use connectivity measures that are insensitive to volume conduction. Examples of such measures are the imaginary coherence, the lagged coherence, and the (weighted) phase-lag index. Their insensitivity to volume conduction stems from an invariant property and it is of both practical and theoretical interest to identify all measures with this property. In this study we derive a set of invariant connectivity measures that are fundamental in the sense that all others can be constructed from them by combination. These ”building blocks” of connectivity measures quantify the lack of invariance of multivariate EEG signals under permutation of the time-points. We use this result to construct a new connectivity measure for stationary aperiodic EEG signals, referred to as the *temporal irreversibility index* (TII) and illustrate its use by applying it to local field potentials recorded from primary visual cortex of a macaque monkey and to EEG data from comatose survivors of cardiac arrest. As far as we are aware, the TII is currently the only functional connectivity measure for aperiodic signals that is insensitive to volume conduction.

## Highlights

• Identify a finite set of fundamental connectivity measures
• Propose the temporal irreversibility index (TII) for aperiodic EEG signals
• TII reflects latency structure in local field potentials
• TII distinguishes good from bad outcomes of comatose patients.

## 1 Introduction

Electroencephalographic (EEG) signals arise from current sources within the brain’s gray matter and are related to these sources by Maxwell’s equations (Hämäläinen et al., 1993). Because a given EEG sensor is sensitive to current sources from multiple locations, interpretation of the results of EEG signal analyses is challenging (Schoffelen and Gross, 2009). This particularly applies to functional connectivity analysis, i.e. statistical dependencies between two or more brain signals (Friston, 2011). Thus observed dependence between the signals measured at two different EEG sensors generally does not imply a dependence between the underlying brain signals (Palva and Palva, 2012). This is referred to as *spurious functional connectivity* due to volume conduction. Invasively recorded electrophysiological signals, such as cortical surface and local field potential recordings, are also subject to spurious connectivity due to volume conduction, although to a lesser extent (Kajikawa and Schroeder, 2011, Buzsáki et al., 2012, Einevoll et al., 2013, Hindriks et al., 2016, Hindriks et al., 2018). Spurious connectivity is one of the main challenges in the analysis of EEG functional connectivity.

A common way to mitigate spurious connectivity is to use measures of functional connectivity that are insensitive to volume conduction. Several such measures have been proposed, such as the imaginary coherence (Nolte et al., 2004), the lagged coherence (Pascual-Marqui, 2007), the (weighted) phase-lag index (Stam et al., 2007a, Vinck et al., 2011), asymmetric phase-modulation functions (Hindriks et al., 2011), and the multivariate interaction measure (Ewald et al., 2012). Given the widespread use of such measures, it is of interest to know the set of all such measures and to be able to construct them in a principled way. More specifically, we are interested in finding a set of connectivity measures that are insensitive to volume conduction and from which all other such measures can be constructed by combination. The measures in this set hence constitute “building blocks” and we will refer to them as *fundamental*. For stationary Gaussian signals in the frequency-domain, fundamental connectivity measures are known (Ewald et al., 2012) but not for arbitrary signals (e.g. non-stationary or non-Gaussian).

In this study, we derive fundamental connectivity measures for arbitrary time-domain EEG signals. These measures can in particular be used to assess functional connectivity between aperiodic EEG signals. We have three reasons to focus on time-domain signals. First, deriving fundamental connectivity measures for frequency-domain signals can be done is a similar way as for time-domain signals and is straightforward. Second, although many insensitive connectivity measures have been proposed for frequency-domain signals, we are not aware of any such measures for time-domain signals. Third, insensitive measures for time-domain signals allow studying functional connectivity in aperiodic EEG signals, which have received increased attention recently (Donoghue et al., 2020).

We derive fundamental measures for bivariate as a well as for higher-order connectivity. The term *higher-order connectivity* refers to statistical dependencies between more than two brain regions. So kth order connectivity refers to the connectivity between k brain regions. Third- and fourth-order connectivity have been observed in spike-train data (Montani et al., 2009, Yu et al., 2011) and have recently received increased attention due to the discovery of discontinuous phase transitions in complex systems with higher-order interactions (Battiston et al., 2021). Recently, several studies have appeared on higher-order connectivity in functional magnetic resonance imaging (MRI) (Faskowitz et al., 2020, Gatica et al., 2021, Luppi et al., 2022, Santoro et al., 2024, Hindriks et al., 2024) as well as EEG data (Herzog et al., 2022, Prado et al., 2023, Hernandez et al., 2024, Ibanez et al., 2024). The connectivity measures that were used in these studies, however, are sensitive to volume-conduction, hence interpretation of the results of their application to EEG data in terms of neuronal communication is unwarranted. The higher-order connectivity measures derived in the current study are insensitive to volume conduction and can thus be applied to EEG data.

In Section 2.1 we discuss the notion of insensitivity to volume conduction as it is used in Nolte et al., 2004, Stam et al., 2007a, Vinck et al., 2011 and other studies. In Section 2.2 we derive a set of fundamental k-order connectivity measures that are insensitive to volume conduction, and in Section 2.3 we illustrate their merit by using them as building blocks to construct a new connectivity measure, which we will refer to as the *temporal irreversibility index* (TII). In Section 3.1 we assess the sampling properties of the TII using simulated signals from an autoregressive model, in Section 3.2 we use a biophysical model of cortical alpha oscillations to show that the TII correlates with axonal projection strength, in Section 3.3 we apply the TII to local field potentials recorded from a Utah array implanted in primary visual cortex of a macaque monkey, and in Section 3.4 we use it to predict the clinical outcome of comatose survivors of cardiac arrest.

## 2 Methods and materials

### 2.1 Connectivity measures insensitive to volume conduction

Functional connectivity is commonly assessed by using measures that are insensitive to volume conduction (Palva and Palva, 2012, Bastos and Schoffelen, 2016). Examples are the imaginary coherence (Nolte et al., 2004), the lagged coherence (Pascual-Marqui, 2007), the (weighted) phase-lag index (Stam et al., 2007a, Vinck et al., 2011), asymmetric phase-modulation functions (Hindriks et al., 2011), and the multivariate interaction measure (Ewald et al., 2012). Their insensitivity to volume conduction is a consequence of the following property (Pascual-Marqui et al., 2018): Let X be an n×k data matrix carrying k EEG signals of length n in its columns and let f be one of the above connectivity measures. Then

**Fig. 1:**
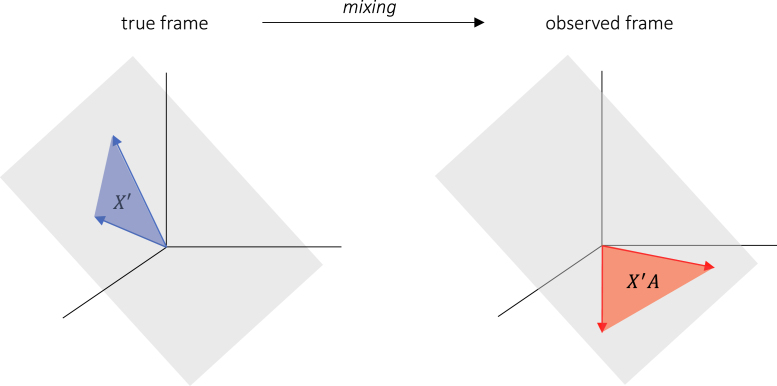
*Effect of mixing on EEG data matrices.* Shown are a true frame $X^{\prime }$ (blue) and the observed frame $X=X^{\prime }A$ (red) in n-dimensional Euclidean space $R^{n}$. The blue and red vectors correspond, respectively, to the true and observed source signals. The true and observed frames span the same k-dimensional subspace (grey plane through the origin) of $R^{n}$.

(1) f(XA)=f(X)

for all invertible k×k matrices A. Bivariate measures correspond to k=2 and multivariate measures to k>2. An example of a k-variate connectivity measure is the multivariate interaction measure (Ewald et al., 2012). We will refer to connectivity measures that satisfy Eq. (1) as *invariant*. From a geometric perspective, invariance means that f only depends on the column space of X and not on the chosen basis (the columns of X). This is illustrated in Fig. 1.

The aim of this study is to derive a set of invariant connectivity measures (for arbitrary k) from which any invariant connectivity measure can be constructed by combination. We will, however, work with a slightly more general notion of invariance, which is motivated by the fact that for hypothesis testing we do not need strict invariance as in Eq. (1). For example, instead of using the imaginary coherence as a test-statistic, we can just as well use the imaginary cross-spectrum (i.e. omit the normalization). But the latter is not strictly invariant, since it satisfies $f\left(XA\right)=\text{det}{\left(A\right)}^{2}f\left(X\right)$. To allow for this, we refer to a connectivity measure f as invariant if

(2) $$f\left(XA\right)=\text{det}{\left(A\right)}^{w}f\left(X\right)$$

for all invertible k×k matrices A and for some integer w called the *weight* of the measure. So the imaginary cross-spectrum in an invariant connectivity measure of weight w=2 and the imaginary coherence and the phase-lag index are invariant connectivity measures of weight 0. Adopting terminology from classical invariant theory (Olver, 2010) we refer to invariant connectivity measures with zero and non-zero weights as *absolute* and *relative*, respectively. Absolute connectivity measures are used to measure the magnitude of neural interactions, whereas relative connectivity measures are used for hypothesis testing for the presence of interactions.

### 2.2 Fundamental connectivity measures for time-domain EEG signals

Let X be an n×k data matrix containing k time-domain EEG signals of length n in its columns. We assume that n≥k and that X has rank k (i.e. the signals are linearly independent). The fundamental connectivity measures of X are defined as the entries of a k-tensor of size n which we will denote by $\wedge^{k}\left(X\right)$ and which is defined as follows. Let $\left(i_{1},\ldots ,i_{k}\right)$ be a strictly increasing sequence of natural numbers between 1 and n and let $X_{i_{1},\ldots ,i_{k}}$ be the matrix obtained from X by selecting rows $i_{1},\ldots ,i_{k}$:

(3) $$X_{I}=\left(\begin{array}{ccc} X_{i_{1},1} & \cdots & X_{i_{1},k}\\ \vdots & \cdots & \vdots \\ X_{i_{k},1} & \cdots & X_{i_{k},k} \end{array}\right).$$

The $\left(i_{1},\ldots ,i_{k}\right)$-th entry of $\wedge^{k}\left(X\right)$ is defined by

(4) $$\wedge^{k}{\left(X\right)}_{i_{1},\ldots ,i_{k}}=\text{det}\left(X_{i_{1},\ldots ,i_{k}}\right)=\underset{\sigma }{\sum }\text{sgn}\left(\sigma \right)X_{i_{\sigma \left(1\right)},1}\cdots X_{i_{\sigma \left(k\right)},k}$$

where σ runs over all permutations of k letters and sgn(σ) denotes the sign of σ. The entries of $\wedge^{k}\left(X\right)$ are called *Plücker coordinates* (Harris, 1992). The tensor $\wedge^{k}\left(X\right)$ has the following property:

(5) $$\wedge^{k}\left(XA\right)=\text{det}\left(A\right)\wedge^{k}\left(X\right)$$

for all invertible k×k matrices A (Munkres, 2018). This shows that its entries are invariants of weight 1 (see Eq. (2)). The fact that all invariant k-variate connectivity measures can be constructed from the entries of $\wedge_{k}\left(X\right)$ by combination, follows from the basic theory of Grassmann manifolds (Harris, 1992). Below we discuss the cases k=2 and k=3 in more detail and provide a functional interpretation of the Plücker coordinates.

Consider the bivariate case (i.e. k=2). For clarity we use different notation and denote the columns of the data matrix X by $x\in R^{n}$ and $y\in R^{n}$. So x and y are observed EEG signals of length n. The 2-tensor $\wedge^{2}\left(x,y\right)$ is given by

(6) $$\wedge^{2}\left(x,y\right)=xy^{⊤}-yx^{⊤}.$$

Its (i,j)-th entry is $\wedge_{ij}^{2}\left(x,y\right)=x_{i}y_{j}-y_{i}x_{j}$. The product $x_{i}y_{j}$ can be thought of as the instantaneous linear interaction between x at time-point i and y at time-point j. Because $y_{i}x_{j}$ is obtained from $x_{i}y_{j}$ by interchanging the time-points i and j, the Plücker coordinate $x_{i}y_{j}-y_{i}x_{j}$ measures the lack of temporal reversibility in the interaction. Thus, a non-zero value of $x_{i}y_{j}-y_{i}x_{j}$ reflects the temporal irreversibility of the interaction at time-points i and j. It is closely related to the time-resolved connectivity measure proposed in Tewarie et al. (2023a).

As an illustration, consider the following simple model for two oscillatory signals. Suppose x and y are harmonic oscillations of the same frequency ω rad/s and are sampled with N samples per second:

$$x_{i}=a_{1}\cos\left(\omega i/N+\phi_{1}\right),$$

and

$$y_{i}=a_{2}\cos\left(\omega i/N+\phi_{2}\right),$$

for i=0,1,2,…,n and where $\phi_{1}$ and $\phi_{2}$ are phases. The Plücker coordinate of (x,y) at time-points (i,i+m) is

$$\wedge^{2}{\left(x,y\right)}_{i,i+m}=a_{1}a_{2}\sin\left(\phi_{1}-\phi_{2}\right)\sin\left(\omega m/N\right).$$

Note that the Plücker coordinate does not depend on i, but only on the lag m=j−i, and that it equals zero if $\phi_{1}=\phi_{2}$ (mod π). So if x and y are in-phase or in anti-phase, the Plücker coordinate is zero and hence it measures the lack of temporal reversibility in the interaction at lag m.

Consider the trivariate case (i.e. k=3). Let $x,y,z\in R^{n}$ be three EEG signals of length n. The Plücker coordinate at time-points i,j,k is

(7) $$\wedge^{3}{\left(x,y,z\right)}_{i,j,k}=\text{det}\left(\begin{array}{ccc} x_{i} & y_{i} & z_{i}\\ x_{j} & y_{j} & z_{j}\\ x_{k} & y_{k} & z_{k} \end{array}\right)=x_{i}y_{j}z_{k}+x_{k}y_{i}z_{j}+x_{j}y_{k}z_{i}-x_{j}y_{i}z_{k}-x_{k}y_{j}z_{i}-x_{i}y_{k}z_{j}.$$

Observe that it is formed by summing triple instantaneous products of the EEG signals at time-points i, j, and k over all six permutations of these time-points, taking into account the signs of the permutations. For example, the product $-x_{j}y_{i}z_{k}$ is obtained from $x_{i}y_{j}z_{k}$ (the original ordering of the signals) by interchanging time-points i and j, which is a permutation with negative sign. This particular form of the Plücker coordinate is what makes it invariant under mixing. A non-zero value reflects the lack of symmetry of the instantaneous linear interaction at time-points i, j, and k under permutation of these time-points and hence is a measure of temporal asymmetry.

### 2.3 The temporal irreversibility index

We use the results from Section 2.2 to construct a new bivariate connectivity measure for stationary time-domain EEG signals, which we will refer to as the *temporal irreversibility index* (TII). Let $x,y\in R^{n}$ be two EEG signals of length n. In Section 2.2 we established that all invariant connectivity measures can be constructed by combining the entries $x_{i}y_{j}-x_{j}y_{i}$ of the anti-symmetric n×n matrix $\wedge^{2}\left(x,y\right)$ (see Eq. (6)). The signed version of the TII of signals x and y is defined as

(8) $$\xi \left(x,y\right)=\frac{1}{M\|\wedge^{2}\left(x,y\right)\|}\overset{M}{\underset{m=1}{\sum }}\overset{n-m}{\underset{i=1}{\sum }}\wedge^{2}{\left(x,y\right)}_{i,i+m}$$

where M is a free parameter which controls the maximum lag between x and y that is taken into account and $\left\|\wedge^{2}\left(x,y\right)\right\|$ denotes the Frobenius norm of $\wedge^{2}\left(x,y\right)$. The temporal irreversibility index ξ(x,y) is an invariant of weight 0, which means that it is strictly invariant under linear mixing of the signals x and y (see Eq. (2)), and can hence be used to quantify functional connectivity. A functional interpretation of the TII can be obtained by expressing it in terms of the crosscorrelation function $r_{m}$ between x and y:

(9) $$\xi \left(x,y\right)=\frac{1}{M\sqrt{1-r_{0}^{2}}}\overset{M}{\underset{m=1}{\sum }}\left(r_{m}-r_{-m}\right)$$

Eq. (9) shows that the TII measures the asymmetry in the crosscorrelation function between x and y and thus quantifies the lack of temporal reversibility of the bivariate signal (x,y). Intuitively, the reason why the TII is insensitive to volume conduction is that volume conduction, being instantaneous, does not alter the temporal ordering of brain activity, and hence can only give symmetric contributions to the crosscorrelation function.

The TII as defined in Eq. (8) is only invariant up to sign (hence the name “signed”). The unsigned version of the TII is defined as

(10) $$\xi^{\prime }\left(x,y\right)=\frac{1}{M\sqrt{1-r_{0}^{2}}}{\left[\overset{M}{\underset{m=1}{\sum }}{\left(r_{m}-r_{-m}\right)}^{2}\right]}^{1/2}.$$

The distinction between the signed and unsigned versions of the TII is similar to that between the imaginary coherence (which is signed) and its absolute value (which is unsigned). Generalization of the TII to k>2 signals is straightforward and the resulting measures can be used for assessing kth order interactions in stationary time-domain EEG signals.

Although a non-zero value of the TII cannot be explained by signal mixing, it is guaranteed to reflect true coupling only if the individual signals are temporally reversible (i.e. if their autocorrelation functions are symmetric) which is necessarily the case if the signals are stationary. However, if one or both of the signals are temporally irreversible, a non-zero value of the TII calculated from the mixed signals could partly reflect this and hence would be spurious. This is in contrast to the Plücker coordinates (Eq. (4)), a non-zero value of which is guaranteed to reflect true coupling (see Appendix B).

## 3 Results

### 3.1 Sampling properties

The TII defined in Section 2.3 is based on the sample autocorrrelation function between the observed EEG signals and consequently exhibits sampling variability. For practical applications, it is important to know its sampling properties, such as its bias and standard error. We assessed these by simulating signals from a second-order bivariate autoregressive process:

(11) $$\left(\begin{array}{c} x_{t}\\ y_{t} \end{array}\right)=\Phi_{1}\left(\begin{array}{c} x_{t-1}\\ y_{t-1} \end{array}\right)+\Phi_{2}\left(\begin{array}{c} x_{t-2}\\ x_{t-2} \end{array}\right)+\left(\begin{array}{c} \epsilon_{t}^{1}\\ \epsilon_{t}^{2} \end{array}\right),$$

where $\Phi_{1}$ and $\Phi_{2}$ are the coefficient matrices at lag 1 and 2, respectively, and $\epsilon_{t}^{1}$ and $\epsilon_{t}^{2}$ are independent zero-mean Gaussian white-noise processes with unit variance. We choose

$$\Phi_{1}=\left(\begin{array}{cc} 0.7 & 0.1\\ 0.1 & 0.7 \end{array}\right),\Phi_{2}=\left(\begin{array}{cc} 0.2 & 0.1\\ -0.1 & 0.2 \end{array}\right).$$

**Fig. 2:**
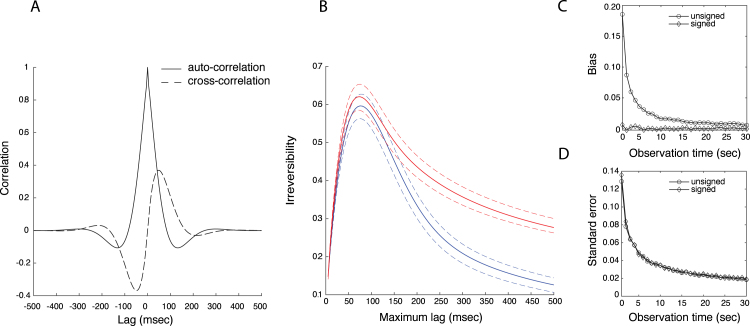
*Sampling properties of the temporal irreversibility index.* A. Theoretical autocorrelation (solid line) and crosscorrelation (dashed line) functions of the autoregressive process. B. Theoretical values of the signed (blue) and unsigned (red) TII, together with the standard errors (dashed lines) of the estimators as functions of the maximum lag. C. Bias of the estimates of the unsigned (circles) and signed (diamonds) TII as a function of observation time. D. Standard error of the estimates of the unsigned (circles) and signed (diamonds) TII as a function of observation time.

Assuming a sampling frequency of 200 Hz, the simulated signals display autocorrelations up to a period of about 300 msec (see Fig. 2A, solid curve). Furthermore, the negative feedback loop at lag 2 gives rise to an asymmetric crosscorrelation function that peaks at a lag of about 50 msec and has a maximal value of about 0.4 (Fig. 2A, dashed curve).

We first assessed the quality of the signed and unsigned TII estimates (Eqs. (9), (10)) as a function of maximum lag M by letting M range from 1 to 100 and computing $10^{4}$ estimates for each value of M, obtained from independent realizations of the autoregressive model. The length of the signals was set to $6\times 10^{3}$, corresponding to an observation time of 30 s. Fig. 2B shows the theoretical values of the signed (solid blue line) and unsigned (solid red line) TII as a function of the maximum lag, together with the standard errors of the respective estimates (dashed lines). The averages of the estimates are not shown as they are practically identical to their theoretical values, showing that for an observation time of 30 s, the estimators are unbiased. The figure shows that the standard errors are practically zero for small values of M, increase with increasing M, and stabilize for large values of M. Furthermore, because the estimates are approximately Gaussian, the associated confidence intervals exclude zero for all values of M, showing that the TII detects the interaction for all values of M.

We next assessed the quality of the estimates as a function of observation time, which ranged from 1 to 30 s in steps of 1 s. For each observation time, we simulated $10^{3}$ pairs of signals with the same model parameters are above, computed the signed and unsigned TII using M=100, and used these to calculate their bias and standard error. The true values of the TII are ξ≈0.13 and $\xi^{\prime }\approx 0.28$. The bias is shown in Fig. 2C. Note that, whereas the estimates for the signed TII are practically unbiased for all observation times, the unsigned TII is severely overestimated for short observation times (approximately up to about 10 s). This is due to the presence of the square in Eq. (10). Fig. 2D shows the standard errors as a function of observation time, illustrating that the standard errors of both the signed and unsigned TII are inversely proportional to observation time, as expected.

### 3.2 Comparison to frequency-domain measures

Since frequency-domain measures, such as the lagged coherence, the imaginary coherence, and the phase lag index, only enable assessing connectivity in oscillatory (i.e. narrowband) signals, comparing their behavior to that of the temporal irreversibility index (TII) can only be done on oscillatory signals. We assess their theoretical behavior as a function of effective coupling strength and type (positive/negative feedback) of the underlying brain regions as well as their standard errors when the measures are estimated from finite signals. We will use simulated signals from a second-order bivariate autoregressive process (Eq. (11)). In all simulations, the maximum lag M in the TII was chosen by computing the TII as a function of M and locating the local maximum with the smallest lag.

In all simulations we choose the parameter values of the two hypothetical brain regions in such a way that they generated oscillatory activity in the absence of effective crossregional coupling:

$$\Phi_{1}=\left(\begin{array}{cc} 0.5 & 0\\ 0 & 0.5 \end{array}\right),\Phi_{2}=\left(\begin{array}{cc} -0.7 & 0\\ 0 & -0.7 \end{array}\right)$$

**Fig. 3:**
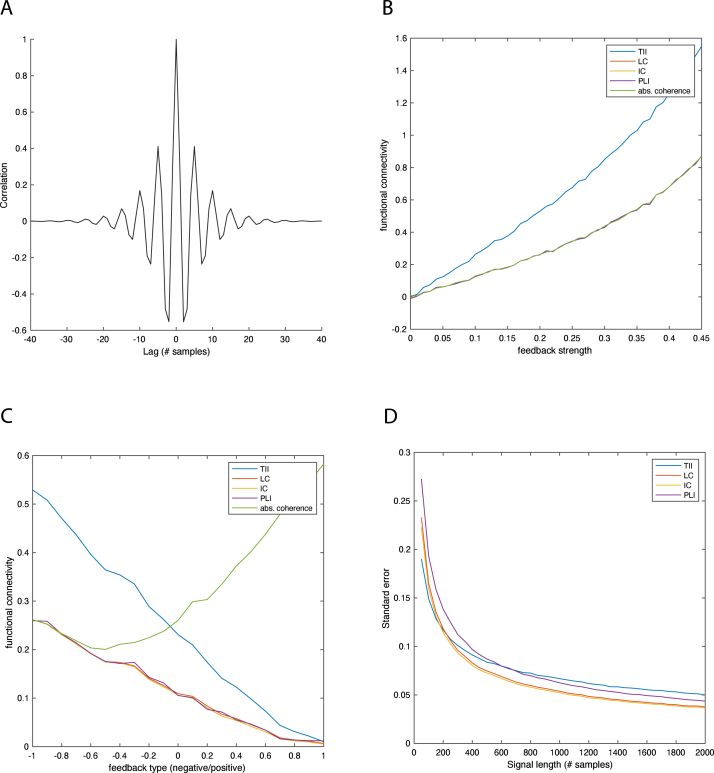
*Comparison to frequency-domain measures.* A. Autocorrelation function of the simulated EEG signals in the absence of effective coupling between the brain regions. B. Functional connectivity (TII, LC, IC, PLI) as a function of the strength of delayed negative feedback. C. Functional connectivity (TII, LC, IC, PLI) as a function of feedback type α (α=−1 corresponds to negative feedback and α=+1 corresponds to positive feedback). D. Standard error as a function of signal-length.

The oscillatory nature of the simulated EEG signals is evident from their autocorrelation functions, which are shown in Fig. 3A.

In the first set of simulations we introduced a delayed negative feedback loop of strength C between the brain regions:

$$\Phi_{2}=\left(\begin{array}{cc} -0.7 & C\\ -C & -0.7 \end{array}\right).$$

Besides amplifying the regions’ oscillatory behavior, the feedback introduced cross-regional coherence between the oscillations. We let C range from 0 to 0.45 and computed the connectivity measures from signals of length $N=10^{6}$ samples. The results are shown in Fig. 3B. We observe that the absolute coherence and all connectivity measures increase as a function of feedback strength. The increase is linear for weak feedback, but becomes non-linear (quadratic) for stronger feedback. We also observe that the TII is roughly proportional to the frequency-domain measures over the entire range of C. This range includes incoherent oscillations (for C=0) as well as strongly coherent ones with absolute coherence of about 0.85 (for C=0.45). These results suggest that assessing functional connectivity in stationary and Gaussian oscillatory EEG signals using the TII, will yield results that are comparable to those obtained by using frequency-domain connectivity measures.

Fig. 3B also makes clear that, over the entire range of the effective connectivity strength C, the imaginary coherence is equal to the absolute coherence and hence the real part of coherence is zero. In other words, the oscillations are 90 degrees out-of-phase. This is why the lagged coherence equals the imaginary coherence in these simulations. The observed equality of the phase-lag index and the lagged coherence is not related to this, but holds for stationary Gaussian signals in general (Nolte et al., 2020, Hindriks, 2021). To assess if the proportionality between the TII and the frequency-domain measures also holds for different phase lags, we gradually shifted the delayed feedback loop from negative to positive. This gradually transforms the oscillations from being 90 degrees out-of-phase to being 180 degrees out-of-phase and hence the frequency-domain measures gradually decrease to zero. Specifically, we choose

$$\Phi_{2}=\left(\begin{array}{cc} -0.7 & 0.2\\ 0.2\alpha & -0.7 \end{array}\right)$$

where the parameter α ranged from −1 (negative feedback) to +1 (positive feedback). Fig. 3C shows the TII and the frequency-domain measures as a function of α. We observe that the frequency-domain measures, as well as the TII, decrease to zero more or less linearly when the feedback shifts from negative to positive. In particular, the TII is more or less proportional to the frequency-domain measures.

In a third set of simulations we compared the standard errors of the TII and the frequency-domain measures when estimated from finite signals. The model parameters were chosen as in the previous simulation with α=0. The signal-length ranged from 50 to 1000 samples. For each signal-length, we simulated $10^{4}$ signals, computed the connectivity measures, and subsequently estimated their standard errors by taking sample standard-deviations. Fig. 3D shows the results. The figure shows that the standard error of the TII is smaller than those of the frequency-domain measures for short signals (less than about 600 samples) and larger for longer (more than about 800 samples) signals. However, the differences are relatively small. Also note that, for all signal lengths, the standard error of the phase-lag index is larger than that of the lagged and imaginary coherence, which is consistent with a recent study using EEG and MEG data (Hindriks, 2021). Lastly, note that the standard error of the lagged coherence is (slightly) higher than that of the imaginary coherence. This is because the lagged coherence requires estimation of both the real and imaginary parts of coherence and both contribute to its sampling variability.

### 3.3 Physiological interpretation

We expect that, for a fixed time delay, the TII reflects the strength of axonal projections, at least to some extent. This is because stronger projections will lead to higher overall (i.e. both instantaneous and lagged) correlations, which increases the area under the crosscorrelation function, thus amplifying its asymmetry. More explicitly, if an increase in axonal projection strength leads to a multiplication of the crosscorrelation function $r_{m}$ by a factor a>0, the TII’s (Eqs. (9), (10)) are scaled by a factor a, and hence linearly depend on a. In addition, the normalization constant $1/\sqrt{1-r_{0}^{2}}$ becomes $1/\sqrt{1-{\left(ar_{0}\right)}^{2}}$, leading to a further (non-linear) increase in irreversibility.

To test this idea, we simulated two thalamo-cortical modules that are connected by directed excitatory inter-cortical axonal projections. The model is described in Hindriks and Tewarie (2023). The inter-cortical delay was set to 50 msec and the projection strength was varied from 0 to 1 in steps of 0.05. For each of the parameter values, the model displayed spontaneous alpha oscillations on top of a broadband 1/f spectral background. To obtain accurate approximations of the crosscorrelation functions, we simulated 500 realizations of one-minute epochs, calculated the sample crosscorrelation functions, and averaged these. This was done for all chosen values of the projection strength. We subsequently calculated the unsigned TII from the averaged crosscorrelation functions. Before calculating the sample crosscorrelation functions, the simulated signals were bandpass filtered in the range 1–40 Hz using a zero-phase fourth-order Butterworth filter.

**Fig. 4:**
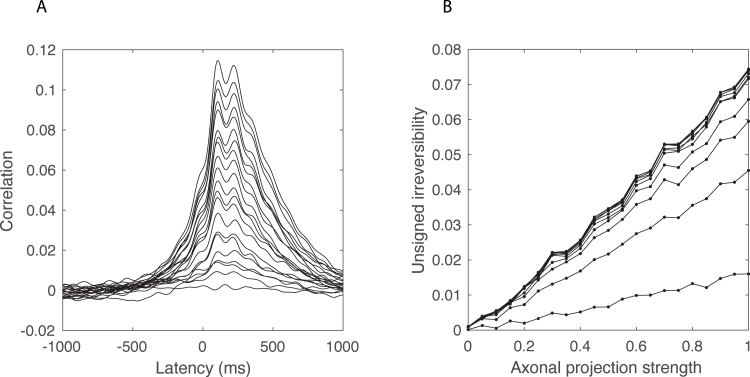
*Temporal irreversibility correlates with axonal projection strength.* A. autocorrelation functions between two uni-directionally coupled thalamo-cortical modules for different inter-cortical axonal projection strengths. B. Temporal irreversibility as a function of axonal projection strength for different values of the maximum lag.

Fig. 4A shows the crosscorrelation functions between the thalamo-cortical modules for different values of the axonal projection strength. Note the presence of a delay at about 50–100 ms for all non-zero values of the axonal projection strength. This delay is due to the combined effects of axonal propagation and synaptic filtering. We also observe that the crosscorrelation increases with increasing axonal projection strength. Fig. 4B shows the unsigned TII as a function of the axonal projection strength for different maximum latencies (ranging from 10 to 100 ms in steps of 10 ms). Note that, for any given value of the maximum latency, irreversibility is proportional to axonal projection strength, thus confirming the above reasoning. These results demonstrate that, at least for unidirectionally coupled thalamo-cortical alpha oscillations, irreversibility is a measure of inter-cortical axonal projection strength.

### 3.4 Application to local field potential data

We used local field potentials recorded with a Utah array implanted in primary visual cortex (V1) of a macaque monkey under resting-state conditions (Chen et al., 2022). We selected the first of 16 Utah arrays that were implanted from recording session 25072017 of monkey L. The array was located in lateral V1 of the left hemisphere, just posterior to the lunate sulcus (see Figure 5A in Chen et al. (2022)). The array comprised an 8 × 8 electrode-grid with 64 iridium oxide electrodes. The length of each electrode shank was 1.5 mm and the spacing between adjacent shanks was 400μm (see Chen et al. (2022) for further details). The monkey was seated in a dark silent room (head-fixed), did not carry out a task, and was allowed to stay awake or fall asleep. Based on recordings of pupil diameter, we selected two epochs during which the monkey’s eyes were open (EO condition) and two epochs during which the monkey’s eyes were closed (EC condition). The durations were 60 s (EO1), 40 s (EO2), 60 s (EC1) and 60 s (EC2). Bad channels were replaced by nearest neighbor averages. The signals were downsampled to 500 Hz and filtered in the range 1–40 Hz with a fourth-order zero-phase Butterworth bandpass filter. The goal of the analysis was to assess differences in the spatial organization of functional connectivity between the EC and EO conditions. For each condition, two epochs were chosen to demonstrate reproducibility of the results.

**Fig. 5:**
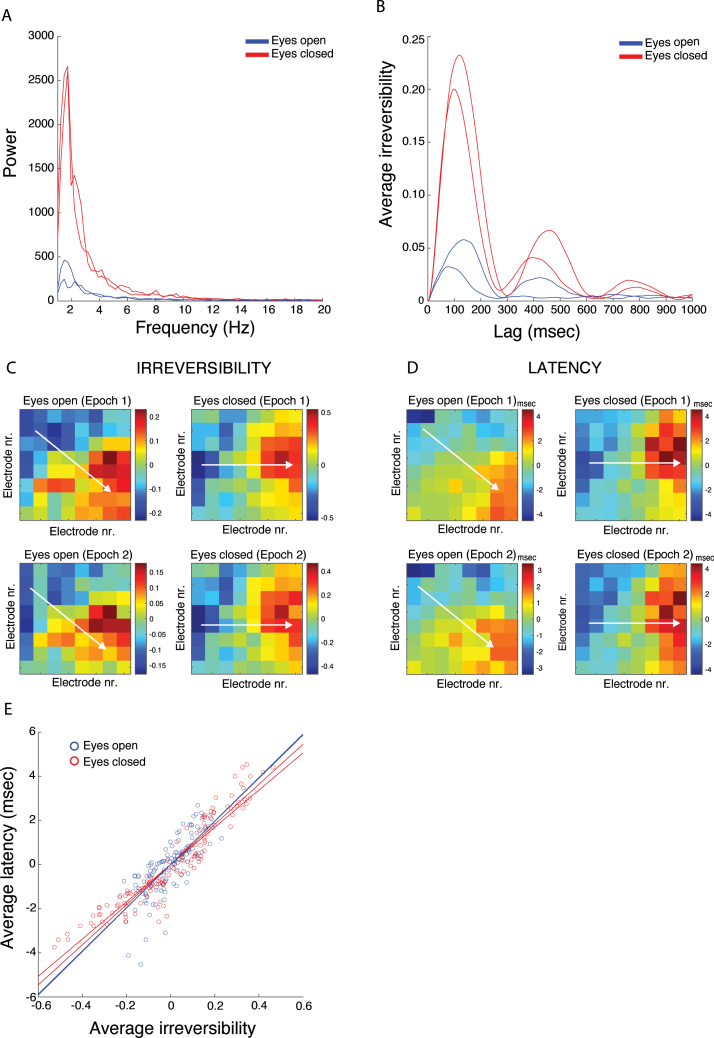
*Temporal irreversibility in resting-state activity of macaque primary visual cortex.* A. Electrode-averaged power spectra of the local field potentials in the eyes-open (blue lines) and eyes-closed (red lines) conditions. The different red/blue lines correspond to different epochs. B. Squared irreversibility as a function of the maximum lag in the eyes-open (blue lines) and eyes-closed (red lines) conditions. The different red/blue lines correspond to different epochs. They were obtained by averaging over electrode-pairs. C. Electrode-averaged irreversibility in the different conditions and epochs. D. Electrode-averaged latencies (in msec) in the different conditions and epochs. E. Scatterplot of electrode-averaged latency against electrode-averaged irreversibility for the eyes-open (blue lines) and eyes-closed (red lines) conditions. The different red/blue lines and circles correspond to different epochs.

Fig. 5A shows the electrode-averaged power spectra in EO (blue) and EC (red) conditions for both epochs. In both conditions we observed broadband activity, which was much stronger in the EC condition. For this application we used the signed version of the TII (i.e. Eq. (9)). To determine the maximum lag M, we inspected the squared TII as a function of lag and averaged it over electrode-pairs. The level of irreversibility clearly depends on the lag (see Fig. 5B). As maximum lags we selected the locations of the local maximum with the smallest latency (136 msec (EO1), 76 msec (EO2), 120 msec (EC1), 100 msec (EC2)). Fig. 5C shows the average irreversibility of each electrode with all other electrodes and thus summarizes the spatial organization of the TII. Absolute irreversibility was higher in the EC condition: The electrode-averaged values are 0.15 (EO1) and 0.10 (EO2) against 0.29 (EC1) and 0.26 (EC2). The figure also makes clear that the spatial patterns of irreversibility are reproducible across epochs and hence are representative for the recording session. In particular, the Pearson correlation between the patterns of the first and second epochs are 0.92 (EO condition) and 0.99 (EC condition).

We also observed a lateral-to-medial gradient in irreversibility in both conditions. However, in the EO condition, the gradient also had an anterior-to-posterior component, which was absent in the EC condition. This is indicated by the white arrows in Fig. 5C. To see if the gradients are related to spatial propagation of the cortical activity, we estimated the latencies between all electrode-pairs by locating the maxima of the respective crosscorrelation functions. Fig. 5D shows, for each electrode, the average latency with all other electrodes. In both conditions, the dominant direction of propagation was from lateral to medial. However, in the EO condition, the propagation also has a component in the anterior-to-posterior direction, and thus closely follows the irreversibility gradient. This is indicated by the white arrows in Fig. 5D. The Pearson correlation between the irreversibility and latency patterns was 0.81 (EO1) and 0.72 (EO2), and 0.95 (EC1) and 0.93 (EC2). Furthermore, the scatter plots in Fig. 5E show that latency is approximately proportional to irreversibility, which can be understood more quantitatively as follows. In Appendix A we derive that, if the crosscorrelation function of a pair of signals peaks at latency $\tau_{0}$ and is symmetric about $\tau_{0}$, the signed TII can be approximated as

$$\xi \approx \frac{2r_{0}\tau_{0}}{\tau_{m}\sqrt{1-r_{0}^{2}}}$$

where $\tau_{m}$ is the maximal lag taken into account in computing the irreversibility index and $r_{0}$ is the instantaneous correlation (i.e. at lag zero) between the signals. Hence the signed irreversibility is proportional to $\tau_{0}$, but the proportionality constant depends on $r_{0}$. However, because in our data the instantaneous correlations are relatively high (overall mean of 0.78) and do not vary much across electrode-pairs (overall standard deviation of 0.17), we expect an approximate linear relation between signed irreversibility and latency, as observed experimentally.

### 3.5 Application to EEG data

A large body of literature has shown that EEG monitoring allows early and reliable prediction of good or poor recovery of comatose patients after cardiac arrest (Cloostermans et al., 2012, Sandroni C, 2022, Hofmeijer and Van Putten, 2016, Spalletti et al., 2016). We include EEG data from hundred patients, as described in our previous work (Tewarie et al., 2023b). All patients underwent continuous EEG monitoring (standard 10–20 system) as part of routine clinical care. Details of data acquisition and inclusion criteria are also reported in Tewarie et al. (2023b). In brief, we included fifty patients with a poor neurological outcome and fifty patients with a good neurological outcome, defined by the cerebral performance category (CPC) score, assessed six months after cardiac arrest. The CPC score quantifies the neurological outcome on a 5-point scale. A CPC=1 (no cerebral damage) or CPC=2 (moderate disability) is generally considered a favorable outcome, while the scores CPC=3 (severe disability), CPC=4 (minimal conscious state or non-responsive sleep-wake syndrome) or CPC=5 (death) reflect a poor neurological outcome.

We selected five minutes of EEG data approximately 48 h after cardiac arrest from patients who exhibited either discontinuous or continuous EEG patterns during this interval. Artifacts were excluded using an automated custom computer algorithm that identified and rejected flat channels, sensor noise, and muscle artifacts (Tjepkema-Cloostermans et al., 2013). In addition, we used independent component analysis (ICA) to identify electrocardiogram artifacts (Hyvarinen, 1999). Every ICA component strongly correlated with the electrocardiogram signal (defined as a Pearson correlation greater than 0.6) was rejected. This resulted in 0–2 rejected ICA components for every subject. Artefact rejection was followed by source reconstruction of the EEG data using *exact low-resolution brain electromagnetic tomography* (eLORETA) (Pascual-Marqui et al., 2006) as implemented in Fieldtrip (Oostenveld et al., 2011). A template T1-weighted image was used to compute a Boundary Element Method (BEM) head model for every subject (Douw et al., 2018). Source reconstructed data were parcellated using the automated anatomical atlas (AAL) (Tzourio-Mazoyer et al., 2002), and time-series within a parcel were averaged to obtain a single time-series per parcel. Only cortical parcels were included for further analysis (Gong et al., 2009), resulting in 78 virtual electrodes. Source reconstructed data were band-pass filtered using a finite impulse response (FIR) filter into the canonical frequency bands: delta (1–4 Hz), theta (4–8 Hz), and alpha (8–13 Hz). For every frequency band, we computed phase synchrony using an existing connectivity measure that is insensitive to signal leakage, i.e. the phase lag index (PLI) (Stam et al., 2007a), lagged coherence (Pascual-Marqui, 2007) and the imaginary coherence (Nolte et al., 2004). In addition, we computed the unsigned TII (Eq. (10)) with a maximal lag of 1 s of the band-pass filtered signals recorded from the virtual electrodes. Statistical testing between groups was performed using t-tests with correction for multiple comparisons using the false discovery rate (Benjamini and Hochberg, 1995).

**Fig. 6:**
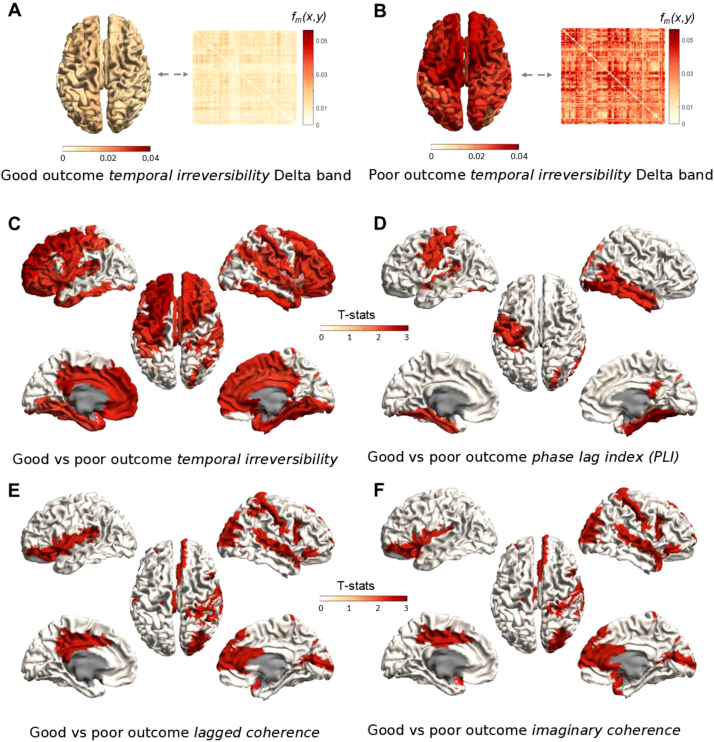
*Temporal irreversibility of EEG signals from comatose survivors of cardiac arrest.* Panels A and B show the average temporal irreversibility matrix across patients in each group. The column average of these matrices is plotted next to them as a brain plot. There is much stronger temporal irreversibility in patients with a poor outcome. Panels C and D show the outcome of statistical testing for the temporal irreversibility and phase lag index, respectively. Panel E and F show the outcome of statistical testing for the lagged coherence and imaginary coherence, respectively. Colors represent t-values for regions that show significant differences between groups (p<0.05 FDR corrected).

Figs. 6A and 6B show the group-averaged irreversibility matrix and corresponding connectivity maps for patients with good and poor outcomes for the delta band respectively. In the poor outcome group, there is overall stronger temporal irreversibility in the delta band across many brain areas. This is also confirmed by formal statistical testing as demonstrated in Fig. 6C, with group differences in the strength of temporal irreversibility especially between left and right temporal and motor areas. Note that these statistically significant group differences were more pronounced than for the phase lag index (PLI), imaginary coherence and lagged coherence for the same data (Fig. 6DEF). We observe that regions where the PLI, imaginary coherence, and lagged coherence showed significant effects, are a subset of the regions significantly different for temporal irreversibility. There were no significantly different results for temporal irreversibility or for the other connectivity metrics for other frequency bands.

## 4 Discussion and conclusions

*Summary and significance.* We introduced a set of invariant measures for time-domain functional connectivity from which all other such measures can be obtained by combination. The fundamental measures can thus be thought of as “building blocks” of invariant connectivity measures. The fundamental measures quantify the lack of invariance of multivariate EEG signals under permutations of time-points and their insensitivity to volume-conduction stems from the fact that volume conduction is instantaneous. Our results hence show that invariance of *any* connectivity measure stems from its invariance under permutations of time-points. We illustrated their use by constructing an invariant connectivity measure for stationary time-domain EEG signals referred to as the *temporal irreversibility index* (TII), which can be used, for instance, for assessing functional connectivity in aperiodic EEG signals. We applied the TII to local field potentials recorded from monkey primary visual cortex and to EEG data recorded from comatose survivors of cardiac arrest. As discussed in more detail below, the approach taken in this study can be further developed and allows defining more general analysis methods for EEG signals that are insensitive to volume conduction.

*Functional interpretation of the TII.* The TII quantifies the extent to which the crosscorrelation function between two EEG signals is asymmetric. If the autocorrelation functions of the individual signals are symmetric, a non-zero value of the TII calculated from two mixed signals reflects the asymmetry of the autocorrelation functions of the true (i.e. unmixed) signals (see Section 2.3). Intuitively, this means that the linear interaction between the true signals has a temporal direction. However, for most EEG signals, a more specific interpretation can be given. Specifically, the asymmetry in the crosscorrelation functions of EEG signals is typically due to a translation of the crosscorrelation function with respect to lag (i.e. a time-delay between the signals) and, therefore, a non-zero value of the TII reflects a non-zero lag between the signals. However, the value of the TII also depends on interaction strength, as measured by the amplitude of the crosscorrelation function. Hence, observed differences in the TII across conditions can generally not be interpreted entirely in terms of lags. This is similar to frequency-domain measures of functional connectivity, such as the lagged coherence, the imaginary coherence, and the phase-lag index, in that they too are functions of both lag and interaction strength. The main difference is that, in the case of frequency-domain measures, the lag and interaction strength are frequency-specific, whereas in the case of the TII, they are defined for aperiodic signals.

*Application to LFP data.* We illustrated the use of the TII by applying it to local field potentials recorded from a Utah array implanted in primary visual cortex of a macaque monkey under eyes-closed and eyes-open resting-state conditions (Chen et al., 2022). Given the aperiodic nature of these signals, their functional connectivity is naturally analyzed in the time-domain. We calculated the average TII between each channel and all other channels and found that the spatial pattern of this averaged TII distinguishes between the eyes-closed and eyes-open conditions. In particular, the averaged TII displayed a spatial gradient, the direction of which differed across the two conditions. Interestingly, the spatial gradients strongly resembled the spatial patterns of latencies between the signals, even though latencies are not invariant. An explicit calculation showed that the TII is indeed approximately proportional to latency for this particular type of signal. This brief application demonstrates that the temporal irreversibility of aperiodic local field potentials, as quantified by the TII, is modulated by behavioral state.

*Application to EEG data.* We also applied the TII to EEG data of comatose survivors of cardiac arrest. We demonstrated that it may be more sensitive to identifying affected brain regions between patients with a good and poor outcome than the phase lag index (Stam et al., 2007b). Remarkably, areas identified using the TII include the subset of regions identified with the phase lag index. Note that our results serve as illustrative purposes only and that an in-depth comparison study is needed to establish if the TII outperforms conventional connectivity measures. While the use of the TII may not only be justified by its insensitivity to signal leakage, it may also be more sensitive to long-distance communication between neuronal populations. Genuine connectivity between neural populations is associated with a conduction delay and a presumed preferred direction of connectivity (see Fig. 6). We note that the TII is related to the irreversibility measure introduced by Deco and coworkers (Deco et al., 2022). In line with previous work, Carrasco-Gómez et al. (2021), we have demonstrated that at 48 h after cardiac arrest, there were only significant differences in temporal irreversibility in the delta band between patients with a good and poor neurological outcome. Presumably, an increased functional connectivity of delta oscillations at this time-point after arrest is associated with poor neurological recovery. Further research in larger clinical cohorts is warranted to study the additional value of the TII in the delta band along existing prognostic EEG features in comatose patients after cardiac arrest.

*Functional versus effective connectivity.* The TII is a measure of functional connectivity in the sense that it vanishes if the signals are statistically independent. It can only be used to detect effective connectivity (i.e. directed information flow or causality) in as far as the latter is reflected in the asymmetry of the signals’ crosscorrelation function. More precisely, the TII can be used to detect effective connectivity in signals for which a reversal of the direction of information flow corresponds to a reversal of the arrow of time and hence to a sign flip of the lag in the signals’ crosscovariance function. An example is a signal that is a delayed copy of another signal plus independent additive noise. Another example is provided by signals that can be modeled by a first-order autoregressive process with uncorrelated noise (see Appendix C for a mathematical proof). Not all signals, however, have this property and hence the TII does not allow for the detection of effective connectivity in general. We note that invariant frequency-domain measures of functional connectivity, such as the lagged coherence (Pascual-Marqui, 2007), the imaginary coherence (Nolte et al., 2004), the (weighted) phase-lag index (Stam et al., 2007a, Vinck et al., 2011), and asymmetric phase-modulation functions (Hindriks et al., 2011), are similarly limited in their ability to infer effective connectivity. Given the limitations of current invariant connectivity measures in detecting causality, an interesting direction for future research is the construction of invariant measures for effective connectivity.

*Geometric interpretation.* We defined the fundamental connectivity measures for k-dimensional EEG signals as the entries of a certain k-dimensional tensor in n-dimensional space (Eq. (4)). These tensors can be given a geometric interpretation that can be exploited to define more properties of EEG signals that are insensitive to volume conduction. Specifically, these tensors have the property that they change sign under any permutation of the k EEG signals. Such tensors are referred to as *alternating* and the collection of all alternating tensors that can be constructed from observed k-dimensional EEG signals form a manifold known as the *Grassmann manifold* (Bendokat et al., 2020). Specifically, the fundamental connectivity measures that can be constructed from observed k-dimensional EEG signals are the coordinates of a point on the Grassmann manifold when it is embedded into the vector space of k-tensors on n-dimensional space (Harris, 1992). This geometric point of view can be exploited as it makes clear that other sets of fundamental measures can be obtained by embedding the Grassmann manifold in different ambient spaces. Furthermore, the Grassmann manifold is naturally endowed with a Riemannian metric and the induced geodesic distances are known in closed form (Batzies et al., 2015) and can be used to define distance measures between observed k-dimensional EEG data-sets that are insensitive to volume conduction. Other analysis methods that can be carried out on Grassmann manifold are linear regression, clustering, and kernel methods (Turaga et al., 2011, Batzies et al., 2015, Zhang et al., 2018). The current study can therefore be regarded as a first step in the systematic development of more comprehensive analysis methods for EEG data that are insensitive to volume conduction.

*Limitations of the TII.* The temporal irreversibility index (TII) is limited in several ways. First, as explained above, the TII cannot be used as a causality measure, unless specific assumptions are made about the EEG signals. We do remark, however, that frequency-domain measures, such as the lagged coherence, the imaginary coherence, and the phase-lag index are also limited in this respect. Second, due to the averaging involved in calculating the TII, the EEG signals are assumed to be stationary. When working with non-stationary signals, one can instead use the Plücker coordinates, which do not require stationarity. Third, as mentioned in Section 2.3, the TII can only be interpreted in terms of functional connectivity if the autocorrelation functions of the EEG signals are symmetric. In other words, the individual EEG signals need to be temporally reversible, which is necessarily the case for stationary signals. If this assumption is not satisfied, the TII calculated from mixed signals can be non-zero, even if the signals are statistically independent. It will be good practice, therefore, to inspect the autocorrelation functions of the EEG signals. We point out, however, that frequency-domain connectivity measures are similarly limited, in that non-stationarity is reflected in non-circularity of the signals’ Fourier coefficients and thus can lead to a non-uniform distribution of relative phases and hence to spurious connectivity (Picinbono, 2002). Lastly, although we have evaluated the performance of the TII on simulated data, these data did not contain sensor noise or (simulated) artifacts. Empirical EEG data, however, are always noise to some extend and might contain artifacts, such as eye-blinks and muscle activity. Further validation of the TII therefore requires investigating the effects of noise and artifacts in both simulated and empirical data.

*Possible extensions.* The methods presented in this study can be extended in several ways. A first possible extension is to frequency-domain EEG signals. We derived building blocks for time-domain EEG signals, but they can be derived for frequency-domain EEG signals as well, by stacking the real and imaginary parts of a complex-valued EEG data matrix. This yields connectivity measures in terms of which all invariant frequency-domain measures, such as the imaginary coherence (Nolte et al., 2004), the lagged coherence (Pascual-Marqui, 2007), the (weighted) phase-lag index (Stam et al., 2007a, Vinck et al., 2011), phase-modulation functions (Hindriks et al., 2011), and the multivariate interaction measure (Ewald et al., 2012), can be expressed. These building blocks also allow generalizing existing frequency-domain measures to non-circular EEG signals (Adali et al., 2011) and can be used to construct time-resolved and higher-order connectivity measures for frequency-domain EEG signals. Another possible extension is to functional connectivity measures for multivariate EEG signals. Connectivity between multivariate signals is referred to as *multidimensional connectivity* and is currently actively explored within the neuroimaging community (Basti et al., 2018, Rahimi et al., 2023). It enables investigating functional connectivity between regions-of-interest, each comprising a (possibly different) number of voxels. The first multidimensional connectivity measure that is invariant under mixing of the signals is the multivariate information measure (Ewald et al., 2012). Within the multivariate context, mixing refers to mixing *within* region-of-interest (intra-regional mixing). The theory presented in the current study can be generalized to multidimensional connectivity measures by considering products of Grassmann manifolds (Ye and Lim, 2016). The building blocks of invariant measures turn out to be symmetric functions of the canonical angles between the EEG data matrices corresponding to the regions-of-interest (Ye and Lim, 2016, Bendokat et al., 2020). Lastly, we mention that the Plücker coordinates and the temporal irreversibility index (TII) could be applied to functional magnetic resonance imaging (fMRI) to assess the latency structure of haemodynamic brain signals and that they complement existing measures (Mitra et al., 2014, Hindriks et al., 2019).

## CRediT authorship contribution statement

**Rikkert Hindriks:** Writing – original draft, Visualization, Software, Investigation, Formal analysis, Conceptualization. **Thomas O. Rot:** Methodology, Formal analysis, Conceptualization. **Michel J.A.M. van Putten:** Software, Investigation, Data curation. **Prejaas Tewarie:** Writing – original draft, Visualization, Software, Investigation.

## Ethics statement

Concerning the LFP data, the original study (Chen et al., 2022) contains the following statement: ”All experimental surgical procedures complied with the NIH Guide for Care and Use of Laboratory Animals (National Institutes of Health, Bethesda, Maryland), and were approved by the institutional animal care and use committee of the Royal Netherlands Academy of Arts and Sciences (approval number AVD- 8010020171046)”. Concerning the EEG data, the institutional review board waived the need for written informed consent as EEG is part of our routine care.

## Declaration of competing interest

The authors declare the following financial interests/personal relationships which may be considered as potential competing interests: Rikkert Hindriks reports financial support was provided by Nederlandse Organisatie voor Wetenschappelijk Onderzoek Utrecht. If there are other authors, they declare that they have no known competing financial interests or personal relationships that could have appeared to influence the work reported in this paper.

## Data Availability

We have shared the link to the data.

## Appendix A Proportionality between temporal irreversibility and latency

For ease of notation we use continuous time instead of discrete time. The arguments, however, are identical, the main difference being the appearance of integrals instead of sums. In continuous time, the signed TII between two signals is

$$\xi =\frac{1}{\tau_{m}\sqrt{1-r{\left(0\right)}^{2}}}\int_{0}^{\tau_{m}}\left(r\left(\tau \right)-r\left(-\tau \right)\right)d\tau .$$

where $\tau_{m}$ is the maximum lag taken into account and r(τ) is the crosscorrelation function between the two signals. Assume that $r\left(\tau \right)=s\left(\tau -\tau_{0}\right)$ for some $\tau_{0}>0$ much smaller than $\tau_{m}$, where the function s is symmetric about 0. Then r peaks at latency $\tau_{0}$ and is symmetric about $\tau_{0}$ and we can therefore write

$$\int_{0}^{\tau_{m}}\left(r\left(\tau \right)-r\left(-\tau \right)\right)d\tau =\int_{0}^{\tau_{m}}r\left(\tau \right)d\tau -\int_{-\tau_{m}}^{0}r\left(\tau \right)d\tau =\int_{0}^{\tau_{m}}r\left(\tau \right)d\tau -\int_{2\tau_{0}}^{2\tau_{0}+\tau_{m}}r\left(\tau \right)d\tau \approx \int_{0}^{\tau_{m}}r\left(\tau \right)d\tau -\int_{2\tau_{0}}^{\tau_{m}}r\left(\tau \right)d\tau =\int_{0}^{2\tau_{0}}r\left(\tau \right)d\tau +\int_{2\tau_{0}}^{\tau_{m}}r\left(\tau \right)d\tau -\int_{2\tau_{0}}^{\tau_{m}}r\left(\tau \right)d\tau =\int_{0}^{2\tau_{0}}r\left(\tau \right)d\tau =\int_{-\tau_{0}}^{\tau_{0}}s\left(\tau \right)d\tau .$$

where, in the second step, we have used the r is symmetric about $\tau_{0}$ and, in the third step, that $t_{0}$ is much smaller than $\tau_{m}$. Because s is symmetric about zero, for small latencies $\tau_{0}$ we have that

$$\int_{-\tau_{0}}^{\tau_{0}}s\left(\tau \right)d\tau \approx 2s\left(0\right)\tau_{0}\approx 2r\left(0\right)\tau_{0}$$

and therefore

$$\xi \approx \frac{2r\left(0\right)\tau_{0}}{\tau_{m}\sqrt{1-r_{0}^{2}}}$$

which shows that the signed irreversibility index is proportional to the delay $\tau_{0}$.

## Appendix B Spurious coupling due to irreversibility of individual EEG signals

Let X be an n×k EEG data matrix, containing k EEG signals of length n in its columns. We treat X as a (zero-mean) random matrix. We first show that a non-zero Plücker coordinate (Eq. (4)) reflects true coupling, irrespective whether the individual signals are temporally irreversible. More formally, this means that, if the signals are statistically independent (i.e. uncoupled), the expected value of all Plücker coordinates of the mixed signals vanishes. Let A be a k×k mixing matrix and let E denote the expectation operator. The expected value of the $\left(i_{1},\ldots ,i_{k}\right)$-th Plücker coordinate of the mixed signals XA is then

$$E\left[\Lambda^{k}{\left(XA\right)}_{i_{1},\ldots ,i_{k}}\right]=E\left[\det\left(A\right)\Lambda^{k}{\left(X\right)}_{i_{1},\ldots ,i_{k}}\right]=\det\left(A\right)E\left[\wedge^{k}{\left(X\right)}_{i_{1},\ldots ,i_{k}}\right]=\det\left(A\right)\underset{\sigma }{\sum }\text{sgn}\left(\sigma \right)E\left[X_{i_{\sigma \left(1\right)},1}\cdots X_{i_{\sigma \left(k\right)},k}\right]=\det\left(A\right)\underset{\sigma }{\sum }\text{sgn}\left(\sigma \right)E\left[X_{i_{\sigma \left(1\right)},1}\right]\cdots E\left[X_{i_{\sigma \left(k\right)},k}\right]=0.$$

Note, however, that the Plücker coordinates do not inform us about *which* of the k signals are coupled. All that can be inferred from observing a non-zero $\left(i_{1},\ldots ,i_{k}\right)$-th Plücker coordinate, is that the k signals are not statistically independent at time-points $i_{1},\ldots ,i_{k}$.

We now show that temporal irreversibility of individual EEG signals can lead to spurious coupling between the mixed signals when using the temporal irreversibility index (TII). We do this for the case of k=2, but a similar argument can be given for any k. Let $x,y\in R^{n}$ be two EEG signals, treated as (zero-mean) random vectors, and let

$$A=\left(\begin{array}{cc} a & b\\ c & d \end{array}\right)$$

be a 2 × 2 mixing matrix. The asymmetric part

$$\gamma_{x^{\prime },y^{\prime }}^{A}\left(\tau \right)=\gamma_{x^{\prime },y^{\prime }}\left(\tau \right)-\gamma_{x^{\prime },y^{\prime }}\left(-\tau \right)$$

of the crosscovariance function $\gamma_{x^{\prime },y^{\prime }}$ between the mixed signals $x^{\prime }=ax+by$ and $y^{\prime }=cx+dy$, can be written as

$$\gamma_{x^{\prime },y^{\prime }}^{A}=ac\gamma_{x,x}^{A}+bd\gamma_{y,y}^{A}+ad\gamma_{x,y}^{A}+bc\gamma_{y,x}^{A}.$$

where $\gamma_{x,x}$ and $\gamma_{y,y}$ are the asymmetric parts of the autocovariance functions of x and y, respectively, and where $\gamma_{x,y}^{A}$ and $\gamma_{x,y}^{A}$ are the asymmetric parts of the crosscovariance functions of the pair (x,y) and (y,x), respectively. This shows that, at any given lag τ, there are two possible causes for an observed asymmetry of the crosscovariance function of the mixed signals, namely, true coupling ($\gamma_{x,y}^{A}$ and $\gamma_{y,x}^{A}$) and temporal irreversibility (at lag τ) of the individual signals ($\gamma_{x,x}^{A}$ and $\gamma_{y,y}^{A}$). In particular, if the signals are statistically independent, the asymmetric part of the crosscovariance function between the mixed signals reduces to

$$\gamma_{x^{\prime },y^{\prime }}^{A}=ac\gamma_{x,x}^{A}+bd\gamma_{y,y}^{A}$$

and hence entirely reflects temporal irreversibility of the individual signals.

## Appendix C Temporal irreversibility of first-order autoregressive processes

Let the discrete-time random variable $X\left(t\right)=\left(X_{1}\left(t\right),X_{2}\left(t\right)\right)$ follow a first-order autoregressive process with uncorrelated noise:

X(t)=AX(t−1)+ϵ(t),

where

$$A=\left(\begin{array}{cc} a_{11} & a_{12}\\ a_{21} & a_{22} \end{array}\right)$$

is the dynamical matrix and $\epsilon \left(t\right)=\left(\epsilon_{1}\left(t\right),\epsilon_{2}\left(t\right)\right)$ is Gaussian noise with covariance matrix $\sigma^{2}I$, where I is the 2 × 2 identity matrix and $\sigma^{2}$ is the variance. The causal structure of the process is characterized by the off-diagonal entries $a_{12}$ and $a_{21}$ of A. Specifically, $a_{12}$ and $a_{21}$ correspond to information flow from $X_{2}$ to $X_{1}$ and from $X_{1}$ to $X_{2}$, respectively. If $a_{12}=a_{21}=0$, there is no information flow, if $a_{12}=0$ or $a_{21}=0$, the flow is unidirectional, and if $a_{21}\neq 0$ and $a_{21}\neq 0$, the flow is bidirectional. When A is replaced by its transpose $A^{⊤}$, the causal structure of the process is “reversed” (e.g. information flow reverses direction). Below we derive that the causal structure of the process is encoded in the asymmetry of its crosscorrelation function $\Sigma \left(l\right)=E\left[X_{t}X_{t+l}\right],$ where l is the lag, and that reversal of the causal structure corresponds to replacing Σ(l) by Σ(−l). A direct consequence is that reversal of the process’ causal structure corresponds to a sign change of the TII.

The autocovariance function of the process can be derived from the Yule–Walker equation and is given by $\Sigma \left(l\right)=A^{l}\Sigma \left(0\right)$ for l=0,1,2,… and $\Sigma \left(-l\right)=\Sigma {\left(l\right)}^{⊤}$. Furthermore, the covariance matrix at lag zero satisfies the discrete-time Lyapunov equation:

$$\Sigma \left(0\right)=A\Sigma \left(0\right)A^{⊤}+\sigma^{2}I.$$

If the spectral radius ρ(A)<1, Σ(0) can be expanded in a power series as

$$\Sigma \left(0\right)=\sigma^{2}\overset{\infty }{\underset{k=0}{\sum }}{\left(AA^{⊤}\right)}^{k}$$

and therefore

$$\Sigma \left(l\right)=\sigma^{2}\overset{\infty }{\underset{k=0}{\sum }}A^{k+l}{\left(A^{⊤}\right)}^{k}$$

for l=0,1,2,…. Now if the process’ causal structure is reversed (i.e. A is replaced by $A^{⊤}$) Σ(l) is replaced by

$$\sigma^{2}\overset{\infty }{\underset{k=0}{\sum }}{\left(A^{⊤}\right)}^{k+l}A^{k}=\sigma^{2}\overset{\infty }{\underset{k=0}{\sum }}{\left(A^{k+l}{\left(A^{⊤}\right)}^{k}\right)}^{⊤}=\Sigma {\left(l\right)}^{⊤}=\Sigma \left(-l\right).$$

Thus, reversal of the process’ causal structure corresponds to replacing Σ(l) by Σ(−l) and it implies that the asymmetric part Σ(l)−Σ(−l) of the autocovariance function, and hence the TII, changes sign upon reversal of the causal structure.
